# Screening for borderline personality disorder after self-harm episodes: validation of the self-rating KABOSS-BD, the borderline subscale of the Karolinska Affective and borderline symptoms scale

**DOI:** 10.1186/s12888-026-08832-1

**Published:** 2026-11-10

**Authors:** Peter Asellus, Sabrina Doering, Ellinor Salander Renberg, Bo Runeson, Margda Waern

**Affiliations:** 1 Department of Clinical Sciences, Unit for Psychiatry, Umeå University, Akutvägen 4 C, Norrlands Universitetssjukhus, Umeå, 907 37 Sweden; 2 Department of Psychiatry and Neurochemistry, Institute of Neuroscience and Physiology, University of Gothenburg, Gothenburg, Sweden; 3 Department of Clinical Neuroscience, Centre for Psychiatry Research, Karolinska Institute, Stockholm, Sweden; 4 Psychosis Clinic, Sahlgrenska University Hospital, Region Västra Götaland, Mölndal, Sweden

**Keywords:** Borderline personality disorder, Emotionally unstable personality disorder, Suicide attempt, Self-harm, Screening, Sex-specific cut-offs, Emergency psychiatric services, Psychometrics

## Abstract

**Background:**

Borderline personality disorder (BPD) is overrepresented among individuals with deliberate self-harm, yet identification of patients requiring diagnostic evaluation remains challenging in emergency settings. Effective screening tools may facilitate earlier identification of symptoms, shorten diagnostic delay, and improve treatment planning. The present study sought to validate the borderline subscale of the Karolinska Affective and Borderline Symptoms Scale (KABOSS-BD) as a self-rating screening tool for BPD in patients presenting with self-harm, and to examine its correlation with clinical diagnosis.

**Methods:**

Data were analysed from 671 patients presenting with self-harm (with or without suicidal intention) at emergency departments in three Swedish cities. We investigated the internal validity of KABOSS-BD and its correlation with clinically diagnosed BPD (ICD-10 Emotionally Unstable Personality Disorder) and determined optimal cut-off scores for women and men for screening purposes.

**Results:**

The KABOSS-BD demonstrated good internal consistency (Cronbach’s α = 0.87) and acceptable diagnostic accuracy in identifying patients with BPD, with an Area Under the Curve (AUC) of 0.73. The optimal cut-off score was 32, yielding sensitivity of 68% and specificity of 68%. Women consistently scored higher than men on the KABOSS-BD, and gender-specific cut-offs improved diagnostic accuracy. The scale performed optimally for women with a cut-off at 33, while for men, the scale performed better at a lower threshold of 26.

**Conclusions:**

The KABOSS-BD demonstrates good internal consistency and acceptable diagnostic accuracy for BPD screening in both women and men who present with suicide attempts and non-suicidal self-harm. The scale’s brevity and ease of administration make it suitable for emergency settings, potentially reducing diagnostic delay and improving treatment allocation for this high-risk population.

**Trial registration:**

Not applicable.

**Supplementary Information:**

The online version contains supplementary material available at https://doi.org/10.1186/s12888-026-08832-1.

## Background

Borderline personality disorder (BPD) is highly prevalent among individuals presenting with suicidal behavior to psychiatric emergency services [1, 2]. Studies of psychiatric inpatients admitted following suicidal behavior have documented substantially elevated rates of BPD when assessed with structured diagnostic interviews [3], underscoring the clinical significance of this association in acute psychiatric settings. Despite this clinical significance, diagnostic delay represents a substantial barrier to effective intervention [4].

This high prevalence of BPD in self-harm populations reflects the disorder’s strong association with suicidal behavior. A recent meta-analysis found that 52% of BPD patients have a lifetime history of suicide attempts, with 80% experiencing suicidal ideation [2]. These rates vary substantially by clinical context, with psychiatric inpatients showing higher suicide attempt rates (75%) compared to outpatients (57%) [2]. The reported lifetime risk of death by suicide in BPD ranges from 3% to 10% [5–7], with this risk further amplified by comorbid mood disorders or substance use disorders [7]. Notably, a 24-year prospective study found that 87.5% of BPD patients who died by suicide had not achieved clinical recovery before death [8], highlighting the critical importance of early, accurate diagnosis for comprehensive risk assessment and timely intervention.

Despite its clinical significance, BPD often remains undiagnosed for extended periods following symptom emergence in adolescence. A coherent syndrome of BPD typically onsets during adolescence, after age 12 years [9]. Retrospective studies indicate that individuals often first contact psychiatric services at ages 17–18, but do not frequently receive an accurate BPD diagnosis at initial presentation [4]. The need for improved recognition is especially pressing in emergency settings, where many individuals with undiagnosed BPD first present in connection with suicide attempts.

To address this diagnostic gap, several screening instruments have been developed for BPD, though few have been specifically validated for emergency settings following suicide attempts or self-harm episodes. Existing tools include the McLean Screening Instrument for BPD (MSI-BPD) [10], which has demonstrated good utility in psychiatric outpatient settings [11], more extensive tools like the Borderline Symptom List (BSL-23) [12], and ultra-brief tools like the 5-item Short-Bord [13]. The Karolinska Affective and Borderline Symptoms Scale - Borderline subscale (KABOSS-BD) [14] represents a potentially valuable addition to these screening tools. With only 9 items, it offers brevity comparable to the shortest existing measures while maintaining comprehensive coverage of key BPD diagnostic criteria. Its integration within the broader KABOSS framework, which includes assessment of depression, anxiety, and obsessive-compulsive symptoms, provides additional context relevant to clinical assessment and treatment planning.

The present study aims to evaluate the psychometric properties and diagnostic accuracy of the KABOSS-BD as a screening tool for BPD in patients presenting at psychiatric emergency departments with self-harm, with or without suicide intent. Specifically, we sought to: assess the internal consistency of the KABOSS-BD in this clinical cohort; to examine the correlation between KABOSS-BD scores and clinical BPD diagnosis; to determine optimal cut-off scores for screening purposes; and to investigate potential gender differences in the scale’s performance.

## Methods

### Study population

This study was conducted at three university hospitals in Sweden (Stockholm, Gothenburg, and Umeå) as part of a prospective multicenter cohort study [15]. Participants were recruited from psychiatric emergency departments in Stockholm and Umeå, and via the psychiatric consultation team in Gothenburg between April 2012 and April 2016.

Eligible participants were patients aged 18 years and above presenting with an episode of deliberate self-harm, which included both suicide attempts (SA) and non-suicidal self-injury (NSSI). Inclusion criteria were a recent episode of self-harm, ability to speak Swedish, and having a Swedish personal identity number (necessary for follow-up in national registers and medical records). Exclusion criteria included conditions that interfered with verbal communication: confusion, severe psychotic symptoms, cognitive impairment, aggressiveness, and intoxication, though patients became eligible if these symptoms abated within a few days of hospital admission. Most research interviews (94%) were conducted within 7 days of the index self-harm episode.

Out of 1,134 eligible patients, 804 (71%) agreed to participate [15]. Of these, 666 (83%) presented with an SA, and 138 (17%) presented with an NSSI, as determined by the Columbia–Suicide Severity Scale [16]. For the current analyses, 133 patients were excluded due to missing KABOSS-BD data, leaving a final sample of 671 participants.

### Diagnostics

Diagnoses were clinical discharge diagnoses established by the treating psychiatrist according to ICD-10 criteria and documented in the patient’s psychiatric medical record. For the purpose of this paper, and for consistency with the broader literature, a participant was considered to have BPD if a clinical diagnosis of Emotionally Unstable Personality Disorder (EUPD, ICD-10 F60.3) was documented in the psychiatric records at the time of the index episode. The validity of EUPD diagnoses in Swedish psychiatric records has been previously established, with a validation study demonstrating 93% agreement for personality disorder diagnoses and 100% confirmation for EUPD diagnoses using ICD-10 criteria.

### Measures

#### KABOSS-BD

The Karolinska Affective and Borderline Symptoms Scale (KABOSS) was administered as part of the research assessment following the index self-harm episode. It is a self-rating instrument designed to capture symptoms associated with common mental disorders including borderline symptoms. We used the original Swedish 9-item version developed by Åsberg and colleagues. KABOSS is an extension of the self-rated Comprehensive Psychopathological Rating Scale for Affective Syndromes [CPRS-S-A; 14] which was developed from the CPRS [17]. The KABOSS has previously been used to assess borderline symptoms in women with a history of suicide attempts [18], demonstrating utility in this clinical population.The complete instrument is composed of four subscales: Depression (the Montgomery-Åsberg Depression Rating Scale self-report, MADRS-S, 9 items), Anxiety (the Brief Scale for Anxiety, 9 items), Obsessive-Compulsive (8 items), and Borderline (9 items). This comprehensive framework allows clinicians to assess multiple symptom domains within a single instrument.

The Borderline subscale (KABOSS-BD) comprises 9 items aligned with the DSM-5 diagnostic criteria for BPD. These items assess:

1. Irritability and anger.
2. Mood instability and mood swings.
3. Ability to understand one’s own feelings.
4. Self-control and impulsivity.
5. Ability to comfort oneself.
6. Feelings of abandonment.
7. Feelings of emptiness.
8. Self-image and identity disturbance.
9. Reality presence (dissociative symptoms).

An English translation of the KABOSS-BD is provided in Additional file 1. KABOSS-BD is a self-rating instrument. Item selection prioritized experiences that patients can readily recognize and rate in themselves, rather than close correspondence to diagnostic terminology. This is reflected, for example, in items assessing the ability to understand one’s own feelings and the ability to comfort oneself, which capture affective awareness and self-soothing difficulties central to the emotion dysregulation seen in BPD. For the same reason, more externally observable relational patterns, such as instability in interpersonal relationships (a core DSM-5 criterion), are not represented as a separate item, as such patterns can be harder for patients to recognize and rate in themselves than internal affective states. Fear of abandonment, a related experience, is included as a separate item, but the broader relational pattern of interpersonal instability itself is not represented on the scale.

All KABOSS subscales are self-rated on a scale from 0 to 6 according to increasing symptom severity; the maximum total score of the 9-item KABOSS BD is thus 54.

### Procedures

Research interviews and data collection were performed by trained mental health professionals (psychiatrists, psychologists, and psychiatric nurses). The KABOSS-BD and other rating scales were administered, and information regarding previous psychiatric treatments, symptoms, and demographic data was gathered through interviews and medical records. Clinical diagnoses were extracted from medical records.

### Statistical analyses

Internal consistency of the KABOSS-BD was assessed using Cronbach’s alpha. Inter-item correlations were calculated to examine the relationships between individual scale items.

For KABOSS, missing data was tested for missingness pattern using Little’s Missing Completely at Random (MCAR) test. In accordance with recommendation by Scheffer [19], assessment scale values identified as MCAR or missing at random (MAR) were imputed by single imputation using the expectation maximum algorithm within the statistical software SPSS. Data was treated as MAR, Little’s MCAR test; X2 (888, *n* = 804) = 988.58, *p* = 0.01. For cases with less than 25% missing data (*n* = 45), values were imputed by single imputation. Cases with 25–100% missing data (*n* = 133) were excluded from the current study.

The relationship between KABOSS-BD scores and clinical BPD diagnosis was analysed using point-biserial correlation. Independent samples t-tests were used to compare KABOSS-BD scores between patients with and without BPD diagnoses, for the total sample, and for men and women separately.

Receiver Operating Characteristic (ROC) curves were constructed and Area Under the Curve (AUC) was calculated to assess the diagnostic accuracy of the KABOSS-BD in identifying BPD. Separate ROC curves were generated for males and females to explore gender-specific accuracy and cut-off scores. Sensitivity, specificity, and Youden’s J statistic (sensitivity + specificity − 1) were calculated for various cut-off scores to identify optimal thresholds for screening purposes. AUC values of 0.70–0.80 were considered acceptable, values 0.80–0.90 were considered excellent, and values above 0.90 outstanding [20]. All tests were two-tailed with significance set at *p* < 0.05.

## Results

### Sample characteristics

The mean age of participants was 37 years (median 32, SD 17, range 18–95). Women were significantly younger than men (mean age 35.8 vs. 40.2 years, *p* = 0.002). Table 1 shows sociodemographic and clinical characteristics of the study sample. Almost one quarter of the women and 5.6% of the men had a clinical EUPD diagnosis (ICD-60.3). The mean KABOSS-BD score was higher in women compared to men.

**Table 1:** Sociodemographic and clinical characteristics of adults presenting with self-harm (with or without suicidal intent) at psychiatric emergency departments

		All (*N* = 671) *N* (%)	Men (*n* = 217) *n* (%)	Women (*n* = 454) *n* (%)	Missing *n* (%)
Current occupation					
Work/student		335 (49.9)	92 (42.4)	243 (53.5)*	--
Unemployed/sick leave/disability pension		335 (49.9)	116 (53.5)	219 (48.2)	--
Retired		59 (8.8)	21 (9.7)	38 (8.4)	--
Living alone		353 (52.6)	133 (61.3)	220 (48.5)*	--
Ongoing treatment for psychiatric problems		490 (73.0)	139 (64.1)	351 (77.3)*	--
Ongoing treatment with antidepressants		447 (66.6)	130 (59.9)	317 (69.8)*	--
Psychiatric inpatient care past 3 months		196 (29.3)	48 (22.1)	148 (32.7)*	1 (0.1)
Suicide attempt at index		551 (82.1)	197 (90.8)	354 (78.0)*	--
Previous suicide attempt^a^		618 (92.2)	200 (92.2)	418 (92.3)	1 (0.1)
Previous non-suicidal self-injury^a^		406 (60.9)	91 (42.1)	315 (69.8)*	5 (0.7)
Psychiatric hospitalization at index^b^		536 (83.5)	171 (83.4)	365 (83.5)	29 (4.3)
ICD-10 diagnosis at index					
Substance use disorders (F10–F19)		310 (46.6)	96 (44.7)	214 (47.4)	6 (0.9)
Psychotic disorders (F20–F29)		19 (2.9)	9 (4.2)	10 (2.2)	6 (0.9)
Mood disorders (F30–39)		253 (38.0)	89 (41.4)	164 (36.4)	6 (0.9)
Anxiety disorders (F40–F48)		275 (41.4)	78 (36.3)	197 (43.8)	6 (0.9)
PTSD (F43.1)		13 (2.0)	--**	--**	--**
Eating disorders (F50)		12 (1.8)	--**	--**	--**
Emotionally unstable personality disorder (F60.3)		119 (17.9)	12 (5.6)	107 (23.8)*	6 (0.9)
Pervasive developmental disorders (F84)		46 (6.9)	13 (6.0)	33 (7.3)	6 (0.9)
ADHD (F90.0)		64 (9.6)	16 (7.4)	48 (10.7)	6 (0.9)
KABOSS subscales		All (*N* = 671) *Mean (SD)*	Men (*n* = 217) *Mean (SD)*	Women (*n* = 454) *Mean (SD)*	
Anxiety		21.9 (9.9)	19.8 (10.4)	22.9 (9.6)*	--
Depression		27.8 (10.7)	25.6 (10.9)	28.8 (10.5)*	--
Obsessive-compulsive		20.7 (9.7)	18.6 (9.9)	21.7 (9.4)*	--
Borderline		26.7 (12.5)	23.1 (12.3)	28.4 (12.3)*	--

^a^according to items CS12a and CS14a respectively, of the Columbia Suicide Severity Rating Scale (C-SSRS) [16]

^b^defined as at least one night’s admission to inpatient care

KABOSS = Karolinska Affective and Borderline Symptoms Scale

*p < 0.05

**Five or fewer individuals

### Internal validity of KABOSS-BD

The KABOSS-BD demonstrated good internal consistency with a Cronbach’s α of 0.87 (ICC = 0.85). Inter-item correlations ranged from 0.40 to 0.50 for all items on the scale (all *p* < 0.001), indicating good cohesion without redundancy.

### Correlation between KABOSS-BD and clinical BPD diagnosis

KABOSS-BD scores were significantly higher (*p* < 0.001) in patients with a BPD diagnosis (*n* = 119, mean 34.7, SD 12.5) compared to those without (*n* = 546, mean 25.0, SD 12.3). This pattern was consistent across genders: women with BPD had a mean score of 35.0 (SD 10.3) compared to women without BPD (M = 26.5 (SD 12.1) *p* < 0.001), while men with BPD had a mean score of 32.4 (SD 9.3) compared to those without BPD (M = 23.1 (SD 12.3), *p* = 0.007). The point-biserial correlation between KABOSS-BD scores and clinical BPD diagnosis was 0.30 (95% CI 0.23–0.36, *p* < 0.001).

### Diagnostic accuracy

**Fig. 1:**
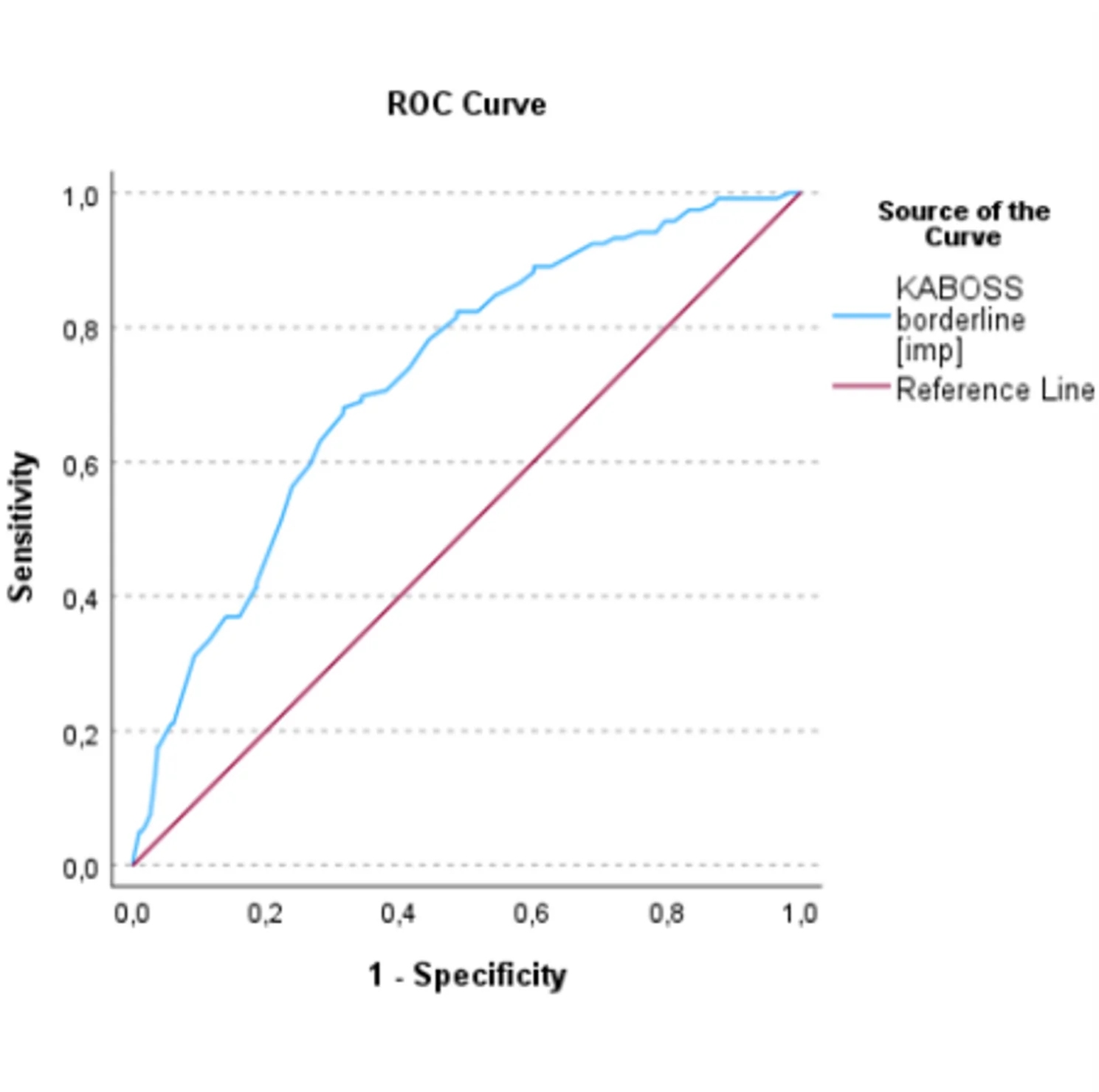
Receiver operating characteristic (ROC) curve for the KABOSS-BD in identifying borderline personality disorder. Note. imp = imputed KABOSS-BD score (see Methods)

The receiver operating characteristic (ROC) curve for the KABOSS-BP in the total sample is shown in Fig. 1. The scale demonstrated acceptable diagnostic accuracy in identifying patients with BPD, with an Area Under the Curve (AUC) of 0.73 (SE = 0.03, 95% CI [0.68, 0.77], *p* < 0.001).

Because imputed values were used in the analysis, optimal cut-off scores are not restricted to whole numbers. The optimal cut-off score based on Youden’s J statistic (0.37) was 31.9, yielding a sensitivity of 68.1% and a specificity of 68.5%. Alternative cut-off points were also examined: a higher cut-off of 32.5 increased specificity to 72.0% but reduced sensitivity to 63.0%, whereas a lower cut-off of 26 increased sensitivity to 82.4% with a specificity of 51.5%.

**Fig. 2:**
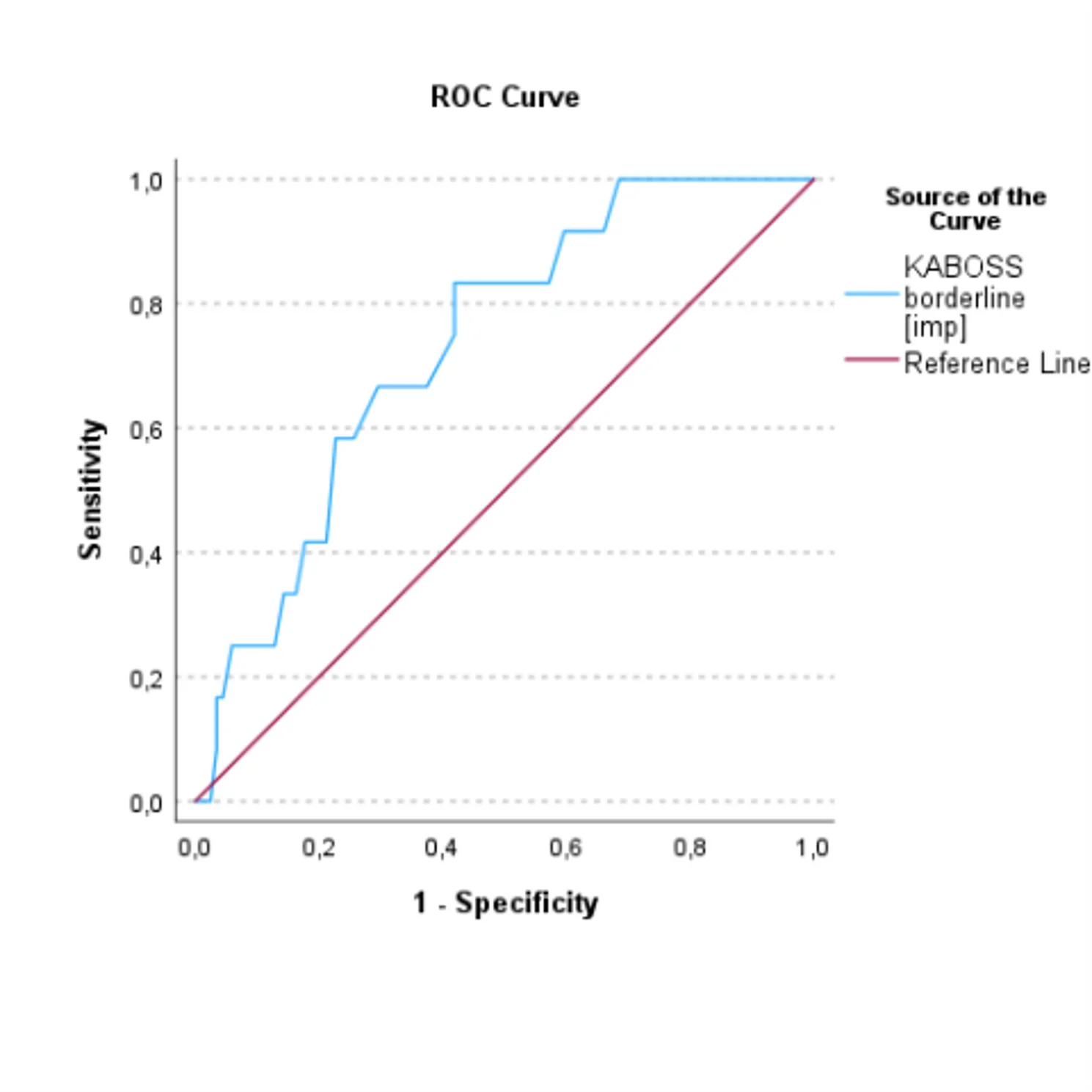
Receiver operating characteristic (ROC) curve for the KABOSS-BD in identifying borderline personality disorder in men. Note. imp = imputed KABOSS-BD score (see Methods)

For men, the AUC was 0.73 (SE = 0.06, 95% CI [0.61, 0.86], *p* < 0.001; Fig. 2). A total score of 26 was identified as the point most correctly distinguishing between those with and without borderline personality disorder. Here, the sensitivity was 83% and the specificity 58%.

**Fig. 3:**
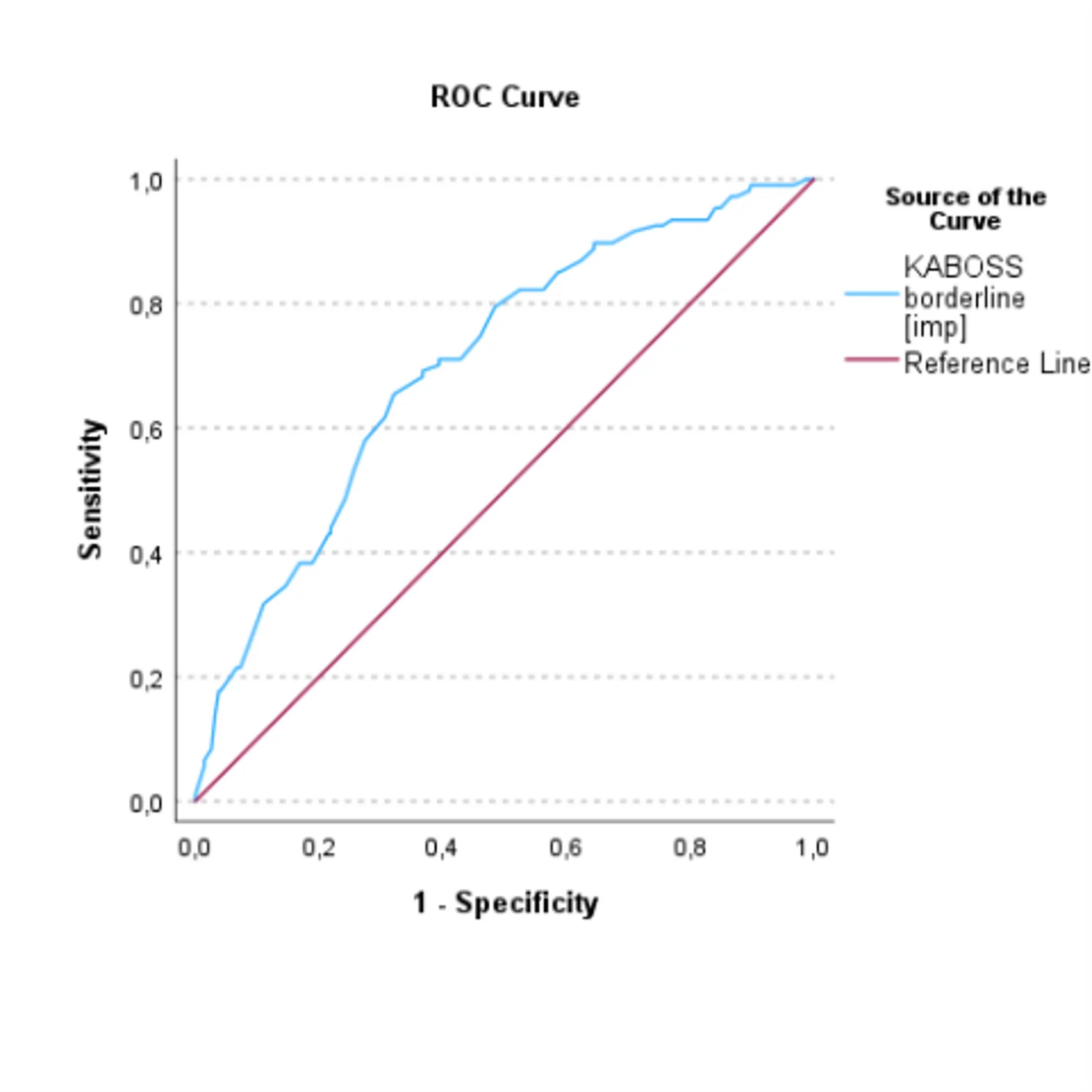
Receiver operating characteristic (ROC) curve for the KABOSS-BD in identifying borderline personality disorder in women. Note. imp = imputed KABOSS-BD score (see Methods)

For females, the AUC was 0.70 (SE = 0.03, 95% CI [0.65, 0.76], *p* < 0.001; Fig. 3), with an optimal cutoff score of 32.5, yielding a sensitivity of 65.4% and a specificity of 67.9% (Youden 0.33).

## Discussion

Results of this study suggest that the KABOSS-BD has potential as a screening tool for BPD in patients presenting with self-harm (with or without suicidal intent) in emergency settings. The scale demonstrates good reliability and acceptable diagnostic accuracy. Women showed higher mean scores and gender-specific cut-off points improved diagnostic accuracy.

The diagnostic accuracy of the scale (AUC = 0.73) represents acceptable discriminative ability for screening measures, providing clinicians with flexibility in choosing cut-off scores based on specific screening objectives. The optimal general cut-off of 32 balances sensitivity (68.1%) and specificity (68.5%), while a lower threshold of 26 substantially increases sensitivity (82.4%), which may be more appropriate when the primary aim is to identify as many possible cases as possible for further assessment. The scale performed optimally for women with a cut-off at 33, while for men, the scale performed better at a lower threshold of 26. The lower cut-off in men likely reflects the previously identified clinical presentations of BPD in men, which tend to be less characterized by depression and anxiety compared to women [21].

The integration of the KABOSS-BD within the broader KABOSS framework represents another advantage. By simultaneously assessing depression, anxiety, obsessive-compulsive symptoms, and borderline traits, clinicians gain a more comprehensive understanding of the patient’s overall psychopathology. This is particularly valuable in the context of suicidal behavior, as most patients with BPD have comorbid mental disorders, including high rates of anxiety disorders, mood disorders, substance use disorders, and PTSD [9].

While structured diagnostic interviews such as the SCID-II remain the gold standard for personality disorder assessment [3], their administration requires considerable time, specialized training, and patient cooperation—resources that tend to be limited in emergency psychiatric settings. The KABOSS-BD addresses this gap by offering a brief, self-administered screening tool that can be completed in minutes and requires minimal clinician time for scoring and interpretation. This practical advantage is particularly important given that the window for initial assessment following self-harm is often narrow, with patients frequently experiencing acute distress, ambivalence about treatment, or early discharge.

The potential clinical impact of improved BPD screening in emergency settings extends beyond immediate risk assessment. Meta-analytic evidence indicates that approximately half to two-thirds of patients with BPD achieve symptomatic remission within 5–15 years [22]. However, achieving full functional recovery appears more challenging. In a prospective 10-year study by Zanarini et al. [23], most patients (93%) achieved symptomatic remission lasting at least two years, but only half attained full recovery, which required both symptomatic remission and good social and vocational functioning over a two-year period. This distinction between symptomatic improvement and functional recovery underscores the importance of early identification and comprehensive treatment. Effective screening tools like the KABOSS-BD could help bridge the diagnostic gap by facilitating earlier recognition of BPD traits when patients present after suicide attempts or NSSI, which is often their first contact with mental health services. This is particularly important given evidence that shows that recovery status significantly impacts mortality risk, with the large majority of suicide deaths occurring among patients who had not achieved recovery from BPD [8]. Early identification is also important considering the high degree of somatic comorbidity and accelerated biological aging associated with BPD, including evidence of epigenetic aging in women with BPD and suicide attempts [24]. Epigenetic aging refers to a biological age estimated from DNA methylation patterns, which can diverge from a person’s chronological age.

Evidence-based guidelines recommend psychotherapy as first-line treatment for BPD [9, 25, 26], with systematic reviews demonstrating efficacy for several psychological interventions, particularly dialectical behavior therapy and specific psychodynamic approaches, though effects are modest and long-term stability requires further investigation [27]. In a qualitative study, women who had been in treatment for BPD provided perspectives on their pathway to recovery and beyond, stressing that therapies must be adapted to a person’s individual needs; if not, backsliding and chronicity could occur [28]. Timely screening and diagnosis are therefore crucial for the initiation of appropriate treatments. While this study focused on the KABOSS-BD’s screening properties, it is important to emphasize that diagnosis of BPD requires comprehensive clinical assessment beyond screening results. The KABOSS-BD should be viewed as a valuable first step that can alert clinicians to the need for more thorough evaluation, rather than a standalone diagnostic tool.

### Strengths and limitations

This study has several notable strengths. The large sample size with a relatively high participation rate provides robust statistical power for the validation analyses. The multisite design across different geographical regions enhances the external validity and generalizability of our findings beyond single-site studies. Additionally, the study was conducted in real-world emergency psychiatric settings with patients presenting in direct connection with self-harm, which is precisely the clinical context where such screening tools are most needed. Our analysis of gender-specific performance is a particular strength as it addresses an important but often overlooked aspect of BPD screening that has significant implications for clinical application.

Several limitations warrant consideration when interpreting these results. We used the ICD-10 diagnosis of Emotionally Unstable Personality Disorder (F60.3) as a proxy for DSM-5 BPD. Although these classifications have substantial overlap, they are not perfectly equivalent, which may affect the generalizability of our findings to settings using other diagnostic systems. Further, our gold standard relies on the patient’s established clinical diagnosis, rather than a structured diagnostic interview conducted specifically for this purpose. While this approach does reflect real-world clinical practice, and validation studies support the overall accuracy of EUPD diagnoses in Swedish psychiatric records [29], inconsistencies in diagnostic practices across settings or clinicians cannot be entirely ruled out.

Our approach may have resulted in underestimation of true BPD prevalence in our sample. Hayashi et al. [3] found that 56% of psychiatric inpatients admitted with recent suicidal behavior met SCID-II criteria for borderline personality disorder, which is substantially higher than the 17.9% with documented BPD diagnoses in the present study. While cultural differences and variations in healthcare systems between Japan and Sweden may contribute to this discrepancy, it likely also reflects the recognized gap between structured diagnostic assessment and routine clinical diagnosis in acute settings. The substantial proportion of non-BPD participants scoring above our recommended cut-offs (48.5% above cut-off 26) may reflect undiagnosed cases. However, this finding also indicates moderate specificity at lower thresholds, emphasizing the tool’s role as a screening instrument rather than a diagnostic instrument, and the importance of comprehensive clinical assessment for positive screens.

The gender distribution of BPD diagnoses in our sample, while consistent with typical clinical populations, meant that the number of men with BPD in our study was relatively small. This limits the reliability of gender-specific cut-off scores for male patients and suggests that the proposed male-specific thresholds should be interpreted cautiously pending validation in larger samples.

## Conclusions

The KABOSS-BD demonstrates good reliability and acceptable diagnostic accuracy as a screening tool for BPD in patients presenting with self-harm. Gender-specific cut-offs improve performance. The scale’s brevity makes it suitable for emergency settings where comprehensive interviews are not feasible. Future research should focus on validating these findings against structured diagnostic interviews as well as assessment of clinical implementation outcomes.

## Supplementary Information

Below is the link to the electronic supplementary material.

Supplementary Material 1

## Data Availability

The data that support the findings of this study are available from the corresponding author upon reasonable request.

## Abbreviations

AUC: Area Under the Curve
BPD: Borderline personality disorder
BSL-23: Borderline Symptom List
CPRS-S-A: Comprehensive Psychopathological Rating Scale for Affective Syndromes
C-SSRS: Columbia Suicide Severity Rating Scale
EUPD: Emotionally Unstable Personality Disorder
KABOSS: Karolinska Affective and Borderline Symptoms Scale
KABOSS-BD: Karolinska Affective and Borderline Symptoms Scale
MADRS-S: Montgomery-Åsberg Depression Rating Scale self-report
MAR: Missing at Random
MCAR: Missing Completely at Random
MSI-BPD: McLean Screening Instrument for BPD
NSSI: Non-suicidal self-injury
ROC: Receiver Operating Characteristic
SA: Suicide attempt
