# Supplementary material for: Screening for borderline personality disorder after self-harm episodes: validation of the self-rating KABOSS-BD, the borderline subscale of the Karolinska Affective and borderline symptoms scale
Source: BMC Psychiatry. 2026 Nov 10;26:884. doi: 10.1186/s12888-026-08832-1 (PMC13749680; doi:10.1186/s12888-026-08832-1)
Supplement: Supplementary file 1 [file 12888_2026_8832_MOESM1_ESM.pdf]

Karolinska Affective and Borderline Symptom Scale, Self-rating (KABOSS – S)

Borderline subscale (KABOSS – BD), Self-rating*

1. Irritability and anger

This question is about how irritable or angry you have felt inside, regardless of whether you have shown something on the outside or not. Consider, in particular, how easily you have become irritated in relation to what triggered your irritability, and how often and how intensely you have felt angry and irritable. If you are completely unable to have any such feelings, tick the “0”.

0 I don’t feel particularly irritable.
1
2 I am more irritable than usual, but the feeling passes quickly.
3
4 I get angry and provoked more easily than usual. Sometimes I lose control in a way that is not normal for me.
5
6 I often feel completely furious inside and have to make an enormous effort to control myself.

2. Mood instability and mood swings

This question concerns shifts between different types of feelings, such as anger, irritability, grief, low spirits, anxiety, worry, joy, guilt, or shame. These mood swings are not necessarily linked to a particular event or situation. A stable mood rarely involves shifting between different feelings several times a day.

0 My mood is fairly stable, and I rarely shift between different feelings. The fluctuations I experience are rarely difficult to cope with.
1
2 My mood is not very stable. Sometimes I experience mood swings that may be difficult to cope with.
3
4 I experience frequent mood swings. It is difficult to plan ahead since I never know how I will feel.
5
6 My mood fluctuates constantly. The tiniest thing can throw me off balance. These mood swings can be intense and painful, and they often take complete control of me. It's really hard.

3. Ability to understand one's own feelings

This question concerns the ability to understand one's own feelings. If you have this ability, your thoughts and feelings are usually connected in a way that you can understand.

0 I can usually understand my own feelings.

1

2 I sometimes find it difficult to understand my own feelings.

3

4 I often experience intense and unpleasant feelings that I don't understand.

5

6 Most of the time, there is chaos inside me, a jumble of incomprehensible and painful feelings.

4. Self-control and impulsivity

By self-control we refer to the ability to control and check sudden impulses, desires and needs. If you have trouble controlling yourself, this may lead to negative consequences for yourself or for those close to you; for example, if you buy something you can't afford or drive too fast.

0 I can control my impulses without any major difficulty. Sometimes I do whatever comes to mind, but it rarely has negative consequences.

1

2 I can control my impulses with a bit of effort. I often do what comes to mind, but it usually doesn't have any negative consequences.

3

4 I find it very difficult to control my impulses. I often act quickly and without thinking, and this often leads to negative consequences.

5

6 Most of the time, I can't control my impulses at all. I rarely think things through and put myself or those around me at great risk.

5. Ability to comfort oneself

This refers to the ability to comfort oneself when feeling bad and to do so in a way that doesn't involve harming oneself. For example, you could watch a good film, read a book or call a friend to clear your mind and feel better.

0 I usually manage to comfort myself when I feel bad.
1

2 I sometimes manage to comfort myself when I feel bad.

3

4 I rarely manage to comfort myself when I feel bad.

5

6 I don't even try to comfort myself because it wouldn't work anyway.

6. Feelings of abandonment

This refers to feelings of loneliness or abandonment, such as getting extremely upset when you have to part with someone, even if only for a short time, or feeling completely abandoned when someone is late for a planned meeting or doesn't get in touch when you expect them to.

0 I rarely feel abandoned.
1

2 I sometimes feel abandoned.

3

4 I very often feel abandoned.

5

6 I have intense and painful feelings of abandonment or loneliness almost all the time.

7. Feelings of emptiness

This refers to feelings of emptiness or loneliness that arise for no apparent reason. It may sometimes feel like having a big hole inside that cannot be filled. How often do you experience feelings of emptiness?

0 Almost never.
1

2 Rarely.

3

4 Sometimes.

5

6 Often.

8. Self-image

This refers to the inner image or perception that a person has of themselves. If you have a stable image or perception of yourself you know who you really are, even if you behave differently with different people.

0 I know fairly well who I am.
1

2 I have sometimes felt unsure of who I really am.

3

4 I am unsure of who I really am most of the time.

5

6 I don’t know at all who I really am.

9. Reality presence

This refers to the feeling of living in reality, knowing and experiencing that you exist here and now. Losing the sense of reality presence can feel unreal or like numbness. It can also be experienced as being disconnected from oneself and feeling detached from your surroundings or as though you cease to exist for a moment.

0 I have “both feet on the ground” and am present in reality.
1

2 I have occasionally felt as if I am losing my grip on reality, but it has never happened.

3

4 I sometimes lose the feeling of being present in what is happening around me.

5

6 I often feel that I am not present in what’s happening around me. Sometimes I feel as if I have ceased to exist.

*Ratings apply to past 3 days.

English translation of the original Swedish version. Reproduced with permission.

Contact: Emeritus professor Marie Åsberg
Department of Clinical Sciences, Karolinska Institutet/Danderyd Hospital
maria.asberg@ki.se
